# External quality assessment for yaws elimination in low- and middle-income countries using plasmid-based proficiency test items

**DOI:** 10.1371/journal.pntd.0013772

**Published:** 2026-03-13

**Authors:** Claudia Mueller, Serges Tchatchouang, Laud Anthony Basing, Solange Ngazoa-Kakou, Kouadio Aboh Hugues, Becca Louise Handley, Camila G. Beiras, Ivy Brago Amanor, Philippe Ndzomo, Mohammed A. Bakheit, Lisa Becherer, Earnest Njih Tabah, Tania Crucitti, Nadine Borst, Christina Ries, Simone Lueert, Sieghard Frischmann, Helena Gmoser, Emelie Landmann, Aboubacar Sylla, Sylvie Mireille Kouamé-Sina, Daniel Arhinful, Patrick Awondo, Sarah Burl, Emma Michèle Harding-Esch, Adingra Tano, Oriol Mitjà, Sara Eyangoh, Kennedy Kwasi Addo, Michael Marks, Sascha Knauf

**Affiliations:** 1 Institute of International Animal Health/One Health, Friedrich-Loeffler-Institut, Federal Research Institute for Animal Health, Greifswald, Insel Riems, Germany; 2 NTD Platform, Centre Pasteur du Cameroun, Yaoundé, Cameroon; 3 Noguchi Memorial Institute for Medical Research, University of Ghana, Accra, Ghana; 4 Institut Pasteur de Côte d’Ivoire, Abidjan, Côte d’Ivoire; 5 National Program of African Trypanosomiasis Elimination, Abidjan, Côte d’Ivoire; 6 Clinical Research Department, London School of Hygiene and Tropical Medicine, London, United Kingdom; 7 Skin Neglected Tropical Diseases and Sexually Transmitted Infections section, Hospital Universitari Germans Trías i Pujol, Fight Infectious Diseases Foundation, Badalona, Spain; 8 Universitat Autònoma de Barcelona, Bellaterra (Cerdanyola del Vallès), Spain; 9 Mast Diagnostica GmbH, Reinfeld, Germany; 10 Laboratory for MEMS Applications, IMTEK - Department of Microsystems Engineering, University of Freiburg, Freiburg, Germany; 11 National Buruli Ulcer, Leprosy, Yaws and Leishmaniasis Control Program, Ministry of Public Health, Yaoundé, Cameroon; 12 Department of Public Health, Faculty of Medicine and Pharmaceutical Sciences, The University of Dschang, Dschang, West Cameroon; 13 Experimental Bacteriology Unit, Institut Pasteur de Madagascar, Antananarivo, Madagascar; 14 Hahn-Schickard, Freiburg, Germany; 15 Hospital for Tropical Diseases, London, United Kingdom; 16 Faculty of Veterinary Medicine, Justus Liebig University, Giessen, Germany; Yale University School of Medicine, UNITED STATES OF AMERICA

## Abstract

**Background:**

We aimed to establish an external quality assessment (EQA) programme for the yaws eradication campaign that would meet the needs of reference and district-level laboratories in low- and middle-income countries.

**Methodology/Principal Findings:**

We designed proficiency testing items (PTIs) using a plasmid containing gene target sequences for *Treponema pallidum* (*TP*) and *Haemophilus ducreyi* (*HD*). The storage stability of the plasmids under different environmental conditions was then tested. A proficiency testing panel of seven swabs loaded with different concentrations of plasmids in different combinations, as well as human HEK293 cells to simulate the sample background, was prepared and sent to participating reference (RL) and district (DL) laboratories in Ghana, Côte d’Ivoire and Cameroon followed by three rounds of blinded proficiency testing. We tested quantitative real-time PCR (qPCR) performance of reference laboratories and loop-mediated isothermal amplification (LAMP) performance of district laboratories and retested 20% of human field samples at the London School of Hygiene & Tropical Medicine laboratories to further assess qPCR quality.

**Findings:**

PTIs proved to be stable in dry conditions with no significant loss of copy number. Participating laboratories achieved qPCR results with a concordance of 95.0-100.0% (97.7% ± 5.2% (mean±standard deviation ((SD)) with the provider and a concordance of 76.0-100.0% (*TP*: 90.3 ± 13.7% and *HD*: 78.5 ± 7.5% (mean±SD)) for LAMP results, with inconsistencies, particularly in the detection of low *HD* plasmid DNA levels combined with high *TP* plasmid copies. Retesting of field samples resulted in 100% correct *TP* and *HD* sample identification by the African reference laboratories.

**Conclusions/Significance:**

We have developed a functional plasmid-based EQA programme specifically designed to meet the needs of resource-poor settings in the tropics. The programme is suitable as a blueprint for other disease programmes.

## Author summary

Yaws caused by the bacterium *Treponema pallidum pertenue* is a Neglected Tropical Disease and remains a public health challenge in many low- and middle-income countries, where reliable laboratory diagnostics are crucial for successful elimination campaigns. The detection of yaws relays on molecular testing (Nucleic Acid Amplification Tests such as PCR) because serology alone cannot distinguish between yaws and other infections such as syphilis caused by *T. pallidum pallidum*. Furthermore, the pathogen is considered uncultivable, which excludes traditional microbiological techniques and explains the shortage of whole organism material that could potentially be used as proficiency testing items (PTIs). Our study presents the development and evaluation of a plasmid-based external quality assessment (EQA) programme tailored to reference and district-level laboratories in Ghana, Côte d’Ivoire, and Cameroon. By designing PTIs that mimic clinical samples, we ensured robust assessment of both quantitative real-time PCR (qPCR) in reference laboratories and loop-mediated isothermal amplification (LAMP) in district laboratories. Our findings highlight the stability of PTIs under field conditions and the overall high concordance rates between participant laboratories and the provider, reinforcing the feasibility of implementing such an EQA programme in resource-poor settings.

## Introduction

External quality assessment (EQA) is a standard quality assessment process in medical laboratory diagnostics that allows the identification of performance problems [1]. Laboratories in high-income countries generally meet high-level laboratory standards, while laboratories in resource-limited countries face challenges due to insufficient infrastructure and/or training [2]. This is particularly the case for laboratories at the district level. Rapid and reproducible data are needed at all healthcare system levels, especially in last-mile eradication efforts where disease prevalence is low, and other diseases mimic a pathogen’s clinical presence. A good example is human yaws, a disease caused by the bacterium *Treponema pallidum* (*TP*) subsp. *pertenue* (*TPE*) [3] which is currently targeted for eradication [4]. The diagnosis of yaws is challenging due to both the inability to serologically distinguish yaws from infections with the related bacterium *TP* subsp. *pallidum* (*TPA*), which causes syphilis [5] and that yaws-like skin lesions caused by co-occurring *Haemophilus ducreyi* (*HD*) cannot be distinguished macroscopically from lesions caused by *TPE* [6]. Therefore, the diagnosis of yaws requires molecular testing (Nucleic Acid Amplification Tests such as PCR) of lesion materials from skin ulcers. Yet, yaws generally infects poor and marginalised human populations, who often have restricted access to healthcare. Decentralised yaws molecular testing at the district level is therefore a programmatic requirement for rapid diagnosis and treatment of infected patients, especially in remote or isolated areas. These areas often present logistical challenges, and mass treatment with azithromycin carries the risk of introducing macrolide resistance, which can lead to treatment failure [3]. Diagnostics for yaws at all healthcare levels require quality control to ensure an efficient, reliable, and robust system. This system enables reference laboratories to focus more on the confirmation of diagnoses rather than screening samples. During the last-mile yaws eradication efforts, every overlooked infection as the potential to prolong the campaign and increases the risk of failing to achieve its goal.

As part of a multi-country EDCTP-funded project (LAMP4Yaws, RIA2018D-2495) to assess the field accuracy of a new diagnostic test in yaws-endemic areas of Ghana, Côte d’Ivoire, and Cameroon [7–10], we aimed to establish an accompanying EQA programme for the yaws eradication campaign that would meet the needs of reference and district-level laboratories and which could serve as a blueprint for other disease programmes.

## Materials and methods

### Ethics statement

Ethical approval for the LAMP4Yaws study from relevant local, national and international committees of the respective countries was obtained by the London School of Hygiene and Tropical Medicine (LSHTM) as well as national ethics committees in Cote d’Ivoire, Cameroon and Ghana and is published elsewhere [7]. Human samples were exclusively handled either in the country of origin or at the LSHTM. Where applicable, clearance under the Access and Benefit Sharing Regulations (Nagoya Protocol) has been obtained by the LSHTM.

### EQA design

**Fig 1:**
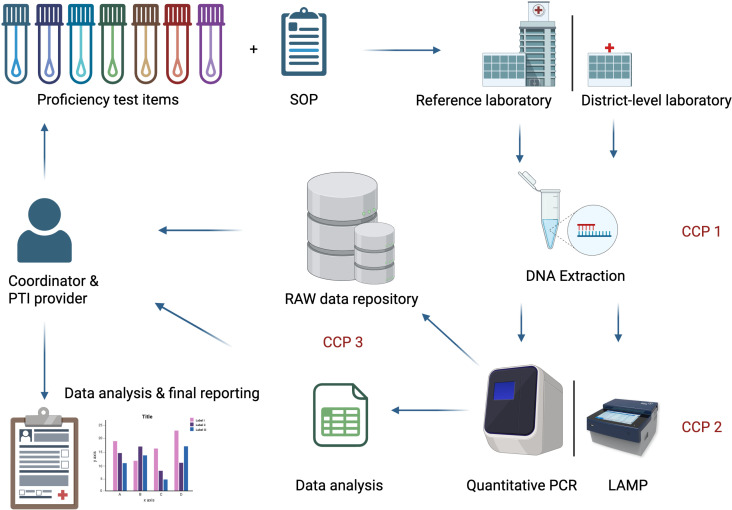
Overview of the EQA programme of the LAMP4Yaws project. CCP = Critical Control Point; CCP1 = DNA extraction control, CCP2 = Validity of molecular analysis at reference laboratory level for qPCR and district-level laboratory for LAMP, CCP3 = Data management and analysis, EQA = External quality assessment, SOP = Standard operating procedure. Created in BioRender. Knauf, S. (2026) http://BioRender.com/d6ya4hv.

We conducted national workshops and stakeholder meetings in Ghana, Côte d’Ivoire and Cameroon where critical control points (CCPs) for quality assessment were identified in the LAMP4Yaws study design [10]. Standard operation procedures (SOPs) were established for participating laboratories with a clear and complete record of the steps carried out. The principles of the World Health Organization (WHO) manual for organising a national EQA programme [11], and the best practice rules [12] were used to build upon the international standard DIN EN ISO/IEC 17043:2023-10 (former 17043:2010-05) for conformity assessment and the general requirements for proficiency testing (PT) [13]. Fig 1 provides an overview of the EQA and CCPs that were overseen by the Friedrich-Loeffler-Institut (FLI).

The EQA programme involved 12 laboratories from Ghana, Côte d’Ivoire, and Cameroon, which participated in the LAMP4Yaws study and conducted testing for *TP* and *HD* in human samples obtained during the study. Details are shown in S1 Table. At the outset, each laboratory provided feedback on its infrastructure and level of staff training. Staff were then trained in good laboratory practice and sample handling using the available equipment and established SOPs. Proficiency testing was performed in three independent rounds using custom control samples described below. Quantitative PCR (qPCR) was considered the gold standard. Consequently, participating reference laboratories (RL) performed qPCR, whereas LAMP assays [14] were performed at the district laboratory level.

FLI sent four Proficiency Test Item (PTI) sets of seven blinded swabs to the respective African reference laboratories in each of the study countries in Africa, which then distributed the PTI sets to the district level. After DNA extraction and molecular testing, all participating laboratories had to report their results in a standardised manner (Fig 1). Briefly, we aimed to achieve an 80%-accuracy in the PT. If a participating laboratory failed to report or the results were not in accordance with the PTI provider’s (FLI) test results, the PT panel had to be retested after a troubleshooting session.

### Design of the proficiency test items

PTIs containing two different plasmid target sequences for the *TP* or *HD* qPCR and LAMP assays were custom-designed using life technologies’ standard shuttle pMA-RQ (AmpR) vector coding for ampicillin resistance (pMA, Origin: *colE1*). The inserted gene target sequences and corresponding sequence data for the *TP polA gene and the variable 8 region of the HD 16SrRNA* gene are shown in S1 Fig and S2 Table. To distinguish between artificial and wild-type gene sequences, M13-tail sequences were added.

For proficiency testing the respective plasmids diluted in 10nM Tris-HCl (pH 8.5) were spiked onto seven FLOQSwabs (COPAN Diagnostics) at defined copy number concentrations in different combinations (Table 1). To simulate the human sample background and to include an extraction control, we complemented each swab with $10^{6}$ human embryonic kidney cells (HEK293, Ref.No. 0197 FLI BioBank). HEK293 cell DNA was verified by *RNAseP* gene detection. Subsequently, swabs were dried at environmental temperature and transferred into Eppendorf DNA LoBind reaction tubes (Eppendorf SE, Germany). Details about plasmid calculation of copy numbers, determination of plasmid concentrations and cell cultivation are given in S1 Fig and S1 Appendix.

**Table 1:** Overview of the proficiency test item panel consisting of seven swabs.

Swab No.	1	2	3	4	5	6	7
*Treponema pallidum* (*TP*)	$10^{9}$	$10^{9}$	$10^{5}$	0	$10^{7}$	0	$10^{5}$
*Haemophilus ducreyi* (*HD*)	$10^{9}$	$10^{5}$	$10^{9}$	0	0	$10^{7}$	$10^{5}$
HEK293 cells	$10^{6}$						

The pMA-RQ (AmpR)-plasmid numbers for *TP* and *HD* represent total copy numbers. HEK293 cells are given as total cell numbers. HEK293 = human embryonic kidney cells.

### Quantitative PCR and loop-mediated isothermal amplification

Following DNA extraction using the QIAamp Mini Kit (Qiagen), qPCRs were performed at African national RLs and the FLI. Likewise, LAMP assays were performed at LSHTM and participating African district laboratories (DLs). For LAMP assays, DNA was extracted with a Mast Isoplex DNA-RNA Extraction Kit (Mast Group GmbH, Germany). In both cases extraction kits were used according to the manufacturer’s protocol with only minor changes. Details are provided in S1 Appendix. Briefly, three single-plex qPCRs were used at the qPCR performing laboratories. Conditions for the *TP polA* [15], *HD 16SrRNA* [14] and the *RNAseP* qPCR [16] are outlined in S1 Appendix and S3-S4 Tables. LAMP assays were run with the MAST ISOPLEX Kit (MAST Diagnostica GmbH, Germany) as published elsewhere [14]. Apart from the retesting (see below), assays were run in triplicates. Further details are provided in S1 Appendix.

### Validation of the proficiency test items

For validation, the PTI provider’s laboratory performed qPCR on a freshly extracted PTI panel using the protocols described in S1 Appendix. We added a standard dilution series of template DNA for *TP polA* and *HD 16SrRNA* plasmids, respectively, in lamda DNA ranging from $10^{6}$ to $10^{1}$ copies/µl. Further, to validate the robustness of our PTIs, we performed storage experiments under different environmental conditions. Briefly, two panels of reaction tubes containing swabs spiked with $10^{6}$ HEK293 cells in combination with either $10^{7}$ (*TP*) or $10^{7}$ (*HD*) plasmid copies were stored at 56°C for 3h or at 37°C in a dry, moist or wet environment for one and three months. Further details are provided in S1 Appendix.

### Proficiency testing

Items were packed, declared and shipped following standards of the International Air Transport Association [17]. The PTIs are not hazardous goods, do not require additional approvals (e.g., Nagoya protocol) or cooling. Three rounds of blinded proficiency testing were conducted. After receiving the blinded PTIs, participating laboratories had 14 days to perform the testing and report their results. Exceptions from this time-frame were granted in case of reasonable request, e.g., when required consumables were missing. Subsequently, the participating laboratories had to share their raw data and an analysis report with the PTI provider.

### Data interpretation and reporting

Data quality was checked by the provider after receiving the raw data and reports from the participating laboratories. In an initial step, the electronically reported results were compared to the corresponding raw data files to rule out major reporting errors or inconsistencies. Any run with a positive negative control was excluded from analysis. Likewise, samples with a negative *RNAseP* reaction (inhibition control) disqualified from inclusion into data analysis. In qPCR, inconsistencies such as outliers in triplicate reactions, standard deviations ≥0.5 of triplicates, high background noise, and non-exponential curves were further investigated and, if necessary, excluded from data analysis. We accepted the exclusion of single outliers in the triplicate reactions in qPCR and LAMP if they were explainable (e.g., due to low copy numbers). LAMP assays were evaluated qualitatively. Swabs were counted as correctly identified if two out of three measurements per swab were in agreement with the panel.

### Retesting of field samples

An extra layer of quality assessment was introduced by retesting 20% of extracted DNA samples (deriving from field samples collected from study participants) at the LSHTM laboratories using qPCR. Samples were selected using a random generator (https://www.dcode.fr/random-sampling), send on dry ice and tested in duplicate for all three targets: *TP polA*, *HD 16SrRNA*, and *RNAseP*.

### Statistics

For measurement of absolute copy numbers of the storage condition experiment, a Kruskal-Wallis-Test with post-hoc Dunn’s test was conducted. For evaluation of proficiency testing, unpaired t-tests were applied. All statistical analysis was conducted using Prism v9.5.1 (GraphPad).

## Results

### Validation of the proficiency test items

**Fig 2:**
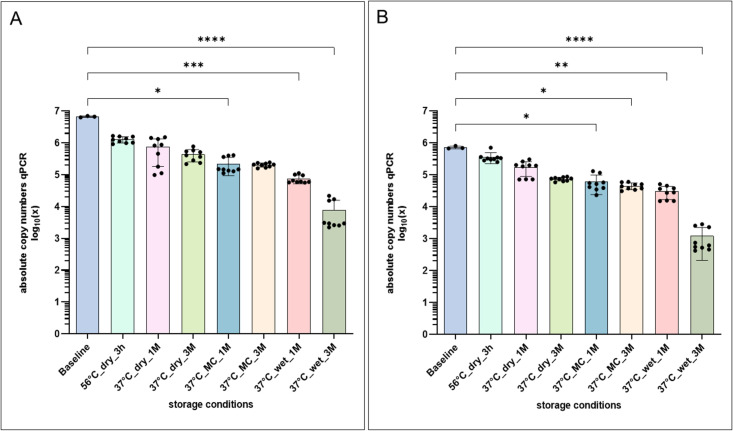
A) qPCR results from the storage experiment for *TP polA* and B) *HD 16SrRNA.* CCP1 = DNA extraction control, CCP2 = Validity of molecular analysis at reference laboratory level for qPCR and district-level laboratory for LAMP, CCP3 = Data management and analysis, EQA = External quality assessment, SOP = Standard operating procedure, CCP = Critical Control Point. Figure created in GraphPad Prism v9.5.1. Datasets are available under https://doi.org/10.5281/zenodo.17398671.

The in-house testing of the extracted PTIs showed an overall two log-scale loss in copy numbers for *TP polA* and *HD 16SrRNA* compared to the actual copy number spiked onto the swabs (S2 Fig). Apart from that, the storage experiment revealed no significant loss of copy numbers for *TP polA* and *HD 16srRN*A in swabs that were stored (short and long-term) under dry conditions. However, copy numbers decreased when PTIs were stored in a moisture chamber for one or three months. The most significant loss of copy number occurred when the swabs encountered a wet environment, regardless of the storage time (Fig 2A-B).

### Proficiency testing

**Fig 3:**
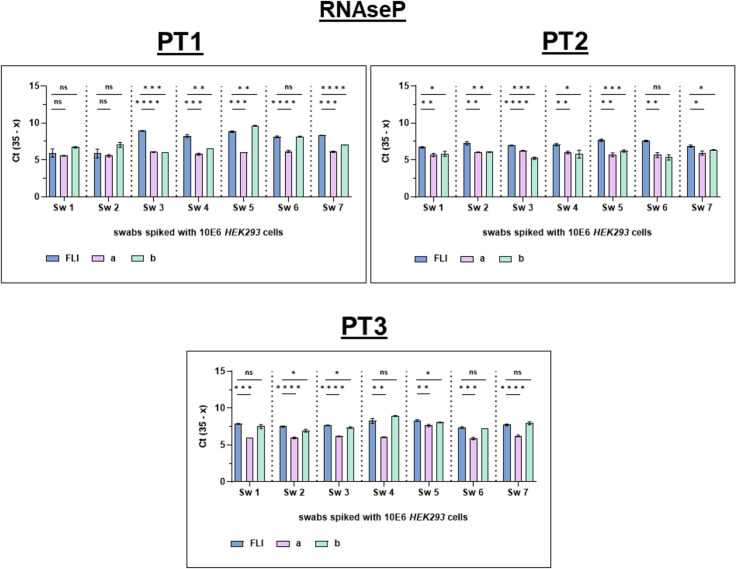
Overview of the qPCR test results of *RNAseP* from all three PT rounds. Blinded panel results were reassorted to the correct order. Swabs were tested in triplicates. One outlier was excluded in the triplicate of PT2 Sw6 laboratory b. FLI = Friedrich-Loeffler-Institut (provider), a = reference laboratory Côte d’Ivoire, b = reference laboratory Cameroon; *RNAseP* = Ribonuclease P, Sw = swab, PT = proficiency test. Figure created in GraphPad Prism v9.5.1. Datasets are available under https://doi.org/10.5281/zenodo.17398671.

**Fig 4:**
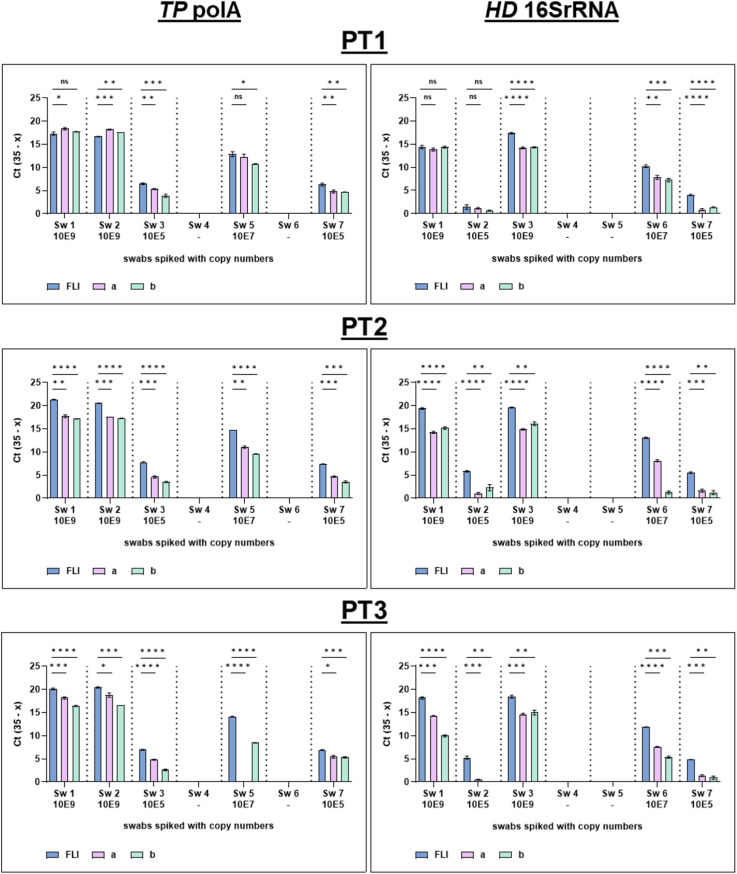
Overview of the qPCR test results of *TP polA* and *HD 16SrRNA* gene from all three PT rounds. Blinded panel results were reassorted to the correct order. Swabs were tested in triplicates. FLI = Friedrich-Loeffler-Institut (provider), a = reference laboratory Côte d’Ivoire, b = reference laboratory Cameroon; *TP* = *Treponema pallidum*; *polA* = polymerase A, *HD* = *Haemophilus ducreyi*, *16SrRNA* = 16S ribosomal ribonucleic acid; Sw = swab; PT = proficiency test. Figure created in GraphPad Prism v9.5.1. Datasets are available under https://doi.org/10.5281/zenodo.17398671.

The PTI provider achieved a qPCR accuracy in proficiency testing of 100.0% in all three PT rounds for all targets, *RNAseP*, *TP polA* and *HD 16SrRNA* (Figs 3 and 4).

Across all PT rounds, two participating RLs successfully amplified the *RNAseP* gene by qPCR (Fig 3). The RL in Ghana had technical problems and was unable to provide raw data and, therefore, was excluded from further evaluation.

The two participating RLs detected the *TP polA* gene and *HD 16SrRNA* gene in all PT rounds using qPCR. Although there was inter-laboratory variation in Ct values, the African RLs agreed with the PTI provider laboratory qualitative results in most cases, with a concordance of 95.0-100.0% (97.7% ± 5.2%) (Fig 4 and S5 Table). In the second PT round, one swab (Sw 6) spiked with medium plasmid conconcentration ($10^{7}$ copy numbers) for the *HD 16SrRNA* gene was identified qualitatively as a low concentration swab ($10^{5}$ copy numbers), and in the third PT round, overall two false negatives were reported for *TP polA* in swab Sw 5 and *HD 16SrRNA* in swab Sw 2. An overall specificity of 100.0% (95% CI: 93.4-100.0) and a sensitivity of 97.8% (95% CI:93.6-99.5) for *TP* and *HD* equally was calculated.

In both Cote d’Ivoire and Cameroon LAMP results were received from one DL. The participating DLs identified the *TP polA* swabs and *HD 16SrRNA* swabs in all PT rounds with a concordance of 90.0%-100.0% (TP; 90.3% ± 13.7%) and 76.0-81.0% (HD; 78.5% ± 7.5%) of all tested PTIs (Table 2). The results were in concordance with the results obtained at LSHTM: 100.0% (*TP*) and 81.0% (*HD*; 81.0% ± 7.1%) (Table 2). We calculated an overall LAMP specificity of 77.8% for *TP* (95% CI: 64.4-88.0) and 90.7% for *HD* (95% CI: 79.7-96.9) as well as a sensitivity of 97.8% for *TP* (95% CI: 93.6-99.5) and 72.6% for *HD* (95% CI: 64.3-79.9).

**Table 2:** LAMP test results for *TP polA* gene and *HD 16SrRNA* gene in all three PT rounds. Blinded panel results were reassorted to the correct order. Results are displayed as correctly identified test results per three replicates. Test results with false positive or false negative outcome are highlighted in bold. LSHTM = The London School of Hygiene & Tropical Medicine; district laboratory Côte d’Ivoire, Yamoussoukro; district laboratory Cameroon, Dimako; Sw = swab; LAMP = Loop-mediated Isothermal Amplification; *TP* = *Treponema pallidum*; *polA* = polymerase A; *HD* = *Haemophilus ducreyi*; *16SrRNA* = 16S ribosomal ribonucleic acid; PT = proficiency test.

Swab No.	1	2	3	4	5	6	7
**LSHTM**							
*TP*	$10^{9}$	$10^{9}$	$10^{5}$	0	$10^{7}$	0	$10^{5}$
*HD*	$10^{9}$	$10^{5}$	$10^{9}$	0	0	$10^{7}$	$10^{5}$
**PT1 - *TP***	3/3	3/3	2/3	3/3	3/3	3/3	3/3
**PT1 - *HD***	3/3	**0/3**	3/3	3/3	3/3	3/3	3/3
**PT2 - *TP***	3/3	3/3	3/3	2/3	3/3	3/3	3/3
**PT2 - *HD***	**0/3**	**0/3**	3/3	3/3	3/3	3/3	3/3
**PT3 - *TP***	3/3	3/3	3/3	2/3	3/3	3/3	3/3
**PT3 - *HD***	3/3	**0/3**	3/3	2/3	3/3	3/3	3/3
Performance % - *TP*	100%						
Performance % - *HD*	81%						
**DL Côte d’Ivoire (Yamoussoukro)**							
*TP*	$10^{9}$	$10^{9}$	$10^{5}$	0	$10^{7}$	0	$10^{5}$
*HD*	$10^{9}$	$10^{5}$	$10^{9}$	0	0	$10^{7}$	$10^{5}$
**PT1 - *TP***	3/3	3/3	3/3	3/3	3/3	3/3	3/3
**PT1 - *HD***	3/3	**0/3**	3/3	3/3	3/3	3/3	3/3
**PT2 - *TP***	3/3	3/3	3/3	**0/3**	3/3	**0/3**	3/3
**PT2 - *HD***	3/3	**0/3**	3/3	**0/3**	3/3	3/3	3/3
**PT3 - *TP***	3/3	3/3	3/3	3/3	3/3	3/3	3/3
**PT3 - *HD***	3/3	**0/3**	3/3	3/3	3/3	3/3	3/3
Performance % - *TP*	90%						
Performance % - *HD*	81%						
**DL Cameroon (Dimako)**							
*TP*	$10^{9}$	$10^{9}$	$10^{5}$	0	$10^{7}$	0	$10^{5}$
*HD*	$10^{9}$	$10^{5}$	$10^{9}$	0	0	$10^{7}$	$10^{5}$
**PT1 - *TP***	3/3	3/3	3/3	3/3	3/3	3/3	3/3
**PT1 - *HD***	**0/3**	**0/3**	3/3	3/3	3/3	3/3	3/3
**PT2 - *TP***	3/3	3/3	3/3	3/3	3/3	3/3	3/3
**PT2 - *HD***	**1/3**	**1/3**	3/3	3/3	3/3	3/3	3/3
**PT3 - *TP***	3/3	3/3	2/3	**1/3**	3/3	**1/3**	2/3
**PT3 - *HD***	2/3	**0/3**	3/3	2/3	3/3	2/3	2/3
Performance % - *TP*	90%						
Performance % - *HD*	76%						

A total of 122 field samples (n = 24 from Cameroon and n = 98 from Côte d’Ivoire) were retested by qPCR at the laboratory in LSHTM. Results indicate a 100% conformity to the test outcomes obtained in Cameroon and Côte d’Ivoire. Further details are provided in S6 Table.

## Discussion

Rapid, reliable, low-cost diagnostics are essential to achieve the WHO goal of eradicating human yaws by 2030. Over the past decade several WHO programmes have significantly improved laboratory capacity strengthening. However, this was usually limited to RLs [18,19]. Our EQA program specifically aimed to meet the needs of RLs and DLs in LMICs. Since early and reliable detection of yaws is crucial for achieving yaws eradication, DLs must be considered the frontline response to detecting new human yaws cases. The need to strengthen district laboratory capacity is recognized by many organisations [20]. Despite this, most DLs are still limited by weak infrastructure, lack of laboratory capacity and undertrained staff [20,21]. Healthcare systems that rely exclusively on RLs for primary testing are largely inefficient. The role of RLs is not to screen all samples from suspected yaws cases but to confirm laboratory results of positively identified cases at the district level.

The lack of standardized materials is a common challenge in EQA programmes [20,21]. Our newly designed EQA programme supports WHO’s mission to improve standards [11] and opens the possibility of increasing the quality of laboratory work at all levels of healthcare. The use of standard shuttle pMA-RQ (AmpR) vectors allows the stable replication of high-copy number plasmids in TOP10 competent *Escherichia coli* (e.g., TOP10 Competent Cells, Invitrogen), an *E*. *coli*-strain engineered for high transformation efficiency. This standard procedure in modern laboratories provides RLs with the option to self-maintain high-quality PTI materials within their institute to ensure a sustainable and ongoing EQA at the national level. Additionally, it replaces the need of cultivation of infectious pathogens with risk of contamination and fluctuating quality and enables RLs to adjust the design of the PTIs according to the local needs and available laboratory infrastructure. While this deviates from DIN EN ISO/IEC 17043:2023-10, which describes conformity assessment and the general requirements for PT, it provides a starting point for EQA in low-resource settings that are not able to perform work at the standard level of a RL.

A major advantage of the newly designed EQA programme is the stability of the PTIs under conditions of increased temperature and humidity. Unstable PT materials often limited PT programmes in the past, e.g., for tuberculosis, malaria and HIV [20,21]. Our swabs can be sent dry to RLs and DLs as we detected no significant degradation of plasmid DNA when stored in a dry environment with an indicated minimum shelf life of three months, sufficient to cover transportation and the usual time delays associated with the organisational challenges of laboratories and environmental conditions in many low-resource settings. Future programmes could also use transformed TOP10 *E*. *coli* to include a technically sounder extraction control, especially if the host background DNA can be neglected.

In this study, the DNA extraction control is based on dried human HEK293 cells. This mimics the human background DNA, that is found in field samples from skin lesions and serves also as an inhibition control for PCR validation. HEK293 cells are an established and easy-to-cultivate cell line [22], but could potentially be replaced by other human cell cultures that can mimic the sample background.

A major challenge for diagnosing pathogens is laboratory contamination with DNA, leading to false positive results [23]. To combat this, we included M13 primer binding sites in the pMA-RQ (AmpR) vectors. This allows an efficient differentiation of laboratory contamination with PTI materials compared to wild-type infection. The latter would be characterised by a negative PCR result using standard M13 primers. Eradication and elimination programmes depend on high-quality standards that can identify false positive test results when prevalence rates are low and declining.

In our newly designed EQA programme, RLs were evaluated according to their qPCR results from three independent PT test rounds. Only two false negative results were seen across all three PT rounds for *TP* and *HD* when the reporting results were compared to the PTI provider’s reference. This is tolerable and indicates the overall high-quality laboratory work that was conducted in the LAMP4Yaws fieldwork in the two successfully participating countries, Cameroon and Côte d’Ivoire. This was further supported by the results obtained from retesting 20% of the field samples at the laboratory in LSHTM.

The LAMP assay included in our EQA program was a promising method for detecting yaws under controlled laboratory conditions [14]. However, its performance under field conditions was unknown at the beginning of the LAMP4Yaws research investigation. LAMP assays have several advantages for use at the DL level. They can be run on inexpensive compact devices with low-cost consumables [7] which makes them practical for use in remote areas where qPCR equipment is unavailable. However, our EQA data further support published findings [7], with the LAMP assay performances being lower than those of the gold standard qPCR.

The LAMP performance data from the EQA highlight that both false positive and false negative test results are a problem. The performances of swab Sw 1 and 2 (both positive in qPCR), which contained high copy numbers for *TP polA* and high- or low-copy numbers for *HD 16SrRNA*, respectively, suggest interferences that inhibit the detection of *HD*. This phenomenon also occurred under high-quality laboratory conditions [14], supporting the need to further revise the *HD LAMP* assay. If LAMP assays are desired, a possible temporal solution would be to run single-plex reactions either as a control or standard procedure whenever high copy numbers for *TP* are suspected. The frequently occurring false positive results in all three PT rounds are also not surprising since LAMP assays tend to have a higher rate of false positive reactions, e.g., due to primer dimerization that causes nonspecific and non-template amplification [24] compared to qPCR.

The main challenge we faced within our EQA programme were organisational issues that are well known to PT programmes in LMICs [21]. These included unavailability of functional laboratory devices, shortage of consumables and data management issues. Broken laboratory equipment and difficult to obtain consumables caused the exclusion of the RLs and DLs in Ghana from our EQA analysis from which no raw data of *RNAseP* results were submitted to the PTI provider. Despite this, the study could still be conducted with the results gained from the remaining two participating countries.

## Conclusion

Special considerations and planning are required to implement EQA programmes in LMICs, where international standard requirements are not easily met. Our plasmid-based EQA programme proved resilient to all conditions that affect laboratories under tropical conditions. It can serve as a blueprint for other disease programmes relevant to human and animal health and avoids the need to deal with permits for infectious materials and negotiations about Access and Benefit Sharing under the Nagoya Protocol.

## Supporting information

S1 Fig Plasmids designed for the EQA programme with gene target inserts for A) *Treponema pallidum* (*TP*) *polA* gene and B) *Haemophilus ducreyi* (*HD*) *16SrRNA* gene. Plasmids were edited with ApE – A plasmid editor - application. bp = base pair; AmpR = Ampicillin resistance.
(PDF)

S2 Fig Validation of the proficiency test items. The loss of copy numbers through proficiency test item extraction can be seen when the actual copy numbers for *TP polA* and *HD 16SrRNA* are compared to the original copy number shown below each bar. The PT numbers represent the round one to three. The bars represent the mean values, with the standard deviation values indicated. Samples were tested in triplicate (each measurement illustrated by a dot). The y-axis indicates the log-scale copy numbers, PT = proficiency test round, Sw = swab.
(PDF)

S1 Appendix Supporting information on materials and methods. (PDF)

S1 Table Participating laboratories in the EQA programme of the LAMP4Yaws project. (PDF)

S2 Table Gene target sequences inserted into the pMA vector. *TP* = *Treponema pallidum*; *polA* = polymerase A; *HD* = *Haemophilus ducreyi.*
(PDF)

S3 Table Primer sequences used for qPCR and 3D digital PCR. *TP* = *Treponema pallidum*, *HD* = *Haemophilus ducreyi*, S = sense, AS = antisense, P = probe.
(PDF)

S4 Table qPCR cycling conditions used by provider (FLI) and African Reference Laboratories for the detection of the (a) *RNAseP*, (b) *TP polA* and (c) *HD 16SrRNA* gene. FLI = Friedrich-Loeffler-Institut (provider), RL = Reference Laboratory.
(PDF)

S5 Table Summary of the qPCR test results from all three PT rounds (correctly identified/total). PT = proficiency test, RL = reference laboratory.
(PDF)

S6 Table Overview of the DNA extracts from field samples that were retested at the London School of Hygiene & Tropical Medicine. *TP* = *Treponema pallidum*; *HD* = *Haemophilus ducreyi*; pos = positive; neg = negative.
(PDF)
