# Supplementary material for: External quality assessment for yaws elimination in low- and middle-income countries using plasmid-based proficiency test items
Source: PLoS Negl Trop Dis. 2026 Mar 13;20(3):e0013772. doi: 10.1371/journal.pntd.0013772 (PMC13035232; doi:10.1371/journal.pntd.0013772)
Supplement: S1 Fig — Plasmids were edited with ApE – A plasmid editor - application. bp = base pair; AmpR = Ampicillin resistance. (PDF) [file pntd.0013772.s001.pdf]

Supporting Information

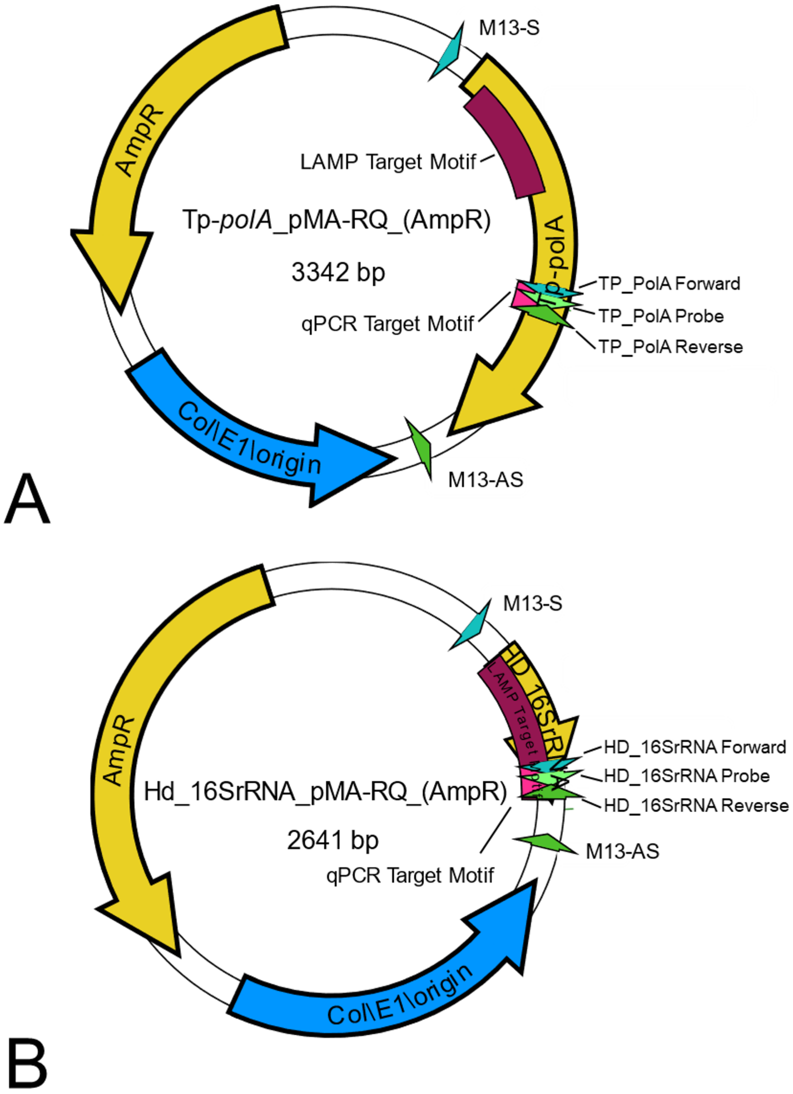

S1 Fig. Plasmids designed for the EQA programme with gene target inserts for A) *Treponema pallidum* (*TP*) *polA* gene and B) *Haemophilus ducreyi* (*HD*) *16SrRNA* gene. Plasmids were edited with ApE – A plasmid editor - application [1]. bp = base pair; AmpR = Ampicillin resistance.

Plasmids were custom designed by Life Technologies using a pMA vector coding for ampicillin resistance (pMA-RQ, Origin: colE1). The vector has a size of 2341bp. The inserted gene target sequences for the *T. pallidum* (*TP*) *polA* gene (1001bp) and the variable 8 region of the *Haemophilus ducreyi* (*HD*) *16SrRNA* gene (300bp) are shown in Fig S1. Corresponding sequence data are presented in S2 Table. This vector is derived from pUC19 but omits unnecessary promoters, enabling biosafety level 1 work. M13 tail

sequences are added to both insert ends for easy differentiation between plasmid contamination and wild-type infection in participating laboratories. The design and ordering of the plasmids were done using the online ordering portal for GeneArt Synthesis Tool (ThermoFisher Scientific). The lyophilized plasmids were diluted in 10nM Tris-HCl (pH 8.5). The plasmid copy numbers were calculated using the formula below and subsequently validated using 3D digital PCR (QuantStudio 3D Digital PCR System, ThermoFisher Scientific).

Calculation of plasmid copy numbers according to Junglen [2]:

$$1 \text{ bp dsDNA} = 660 \, \frac{\text{g}}{\text{mol}} \tag{1}$$

$$1 \text{ mol} = 6.023 \times 10^{23} \text{ mol}^{-1} \quad \text{[Avogadro's Constant]} \tag{2}$$

$$\text{Plasmid size [bp]} = \text{vector size [bp]} + \text{insert size [bp]} \tag{3}$$

$$\text{Plasmid size [bp]} \times 660 \, \frac{\text{g}}{\text{mol}} = \text{molarity} \left[\frac{\text{g}}{\text{mol}}\right] \tag{4}$$

$$\frac{\text{Molarity [g/mol]}}{6.023 \times 10^{23}} = \text{weight [g/plasmid or ng/plasmid]} \tag{5}$$

$$\frac{\text{DNA concentration [ng/}\mu\text{l]}}{\text{weight [ng/plasmid]}} = \text{plasmid copy number [plasmid/}\mu\text{l]} \tag{6}$$

References

1. Davis MW, Jorgensen EM. ApE, A Plasmid Editor: A Freely Available DNA Manipulation and Visualization Program. Front Bioinform. 2022;2:818619.
2. Junglen S. Untersuchungen zur Vektor-und Arbovirenprävalenz in Randzonen des tropischen Regenwaldes des Tai Nationalparks, Côte d'Ivoire und Charakterisierung neuer Virusisolate. Doctoral Thesis. 2007. 1–140 p. Available from: `https://depositonce.tu-berlin.de/items/f2bb22a1-435c-4135-b939-240127990a17`
