# Supplementary material for: External quality assessment for yaws elimination in low- and middle-income countries using plasmid-based proficiency test items
Source: PLoS Negl Trop Dis. 2026 Mar 13;20(3):e0013772. doi: 10.1371/journal.pntd.0013772 (PMC13035232; doi:10.1371/journal.pntd.0013772)
Supplement: S2 Fig — The loss of copy numbers through proficiency test item extraction can be seen when the actual copy numbers for TP polA and HD 16SrRNA are compared to the original copy number shown below each bar. The PT numbers represent the round one to three. The bars represent the mean values, with the standard deviation values indicated. Samples were tested in triplicate (each measurement illustrated by a dot). The y-axis indicates the log-scale copy numbers, PT = proficiency test round, Sw = swab. (PDF) [file pntd.0013772.s002.pdf]

Supporting Information

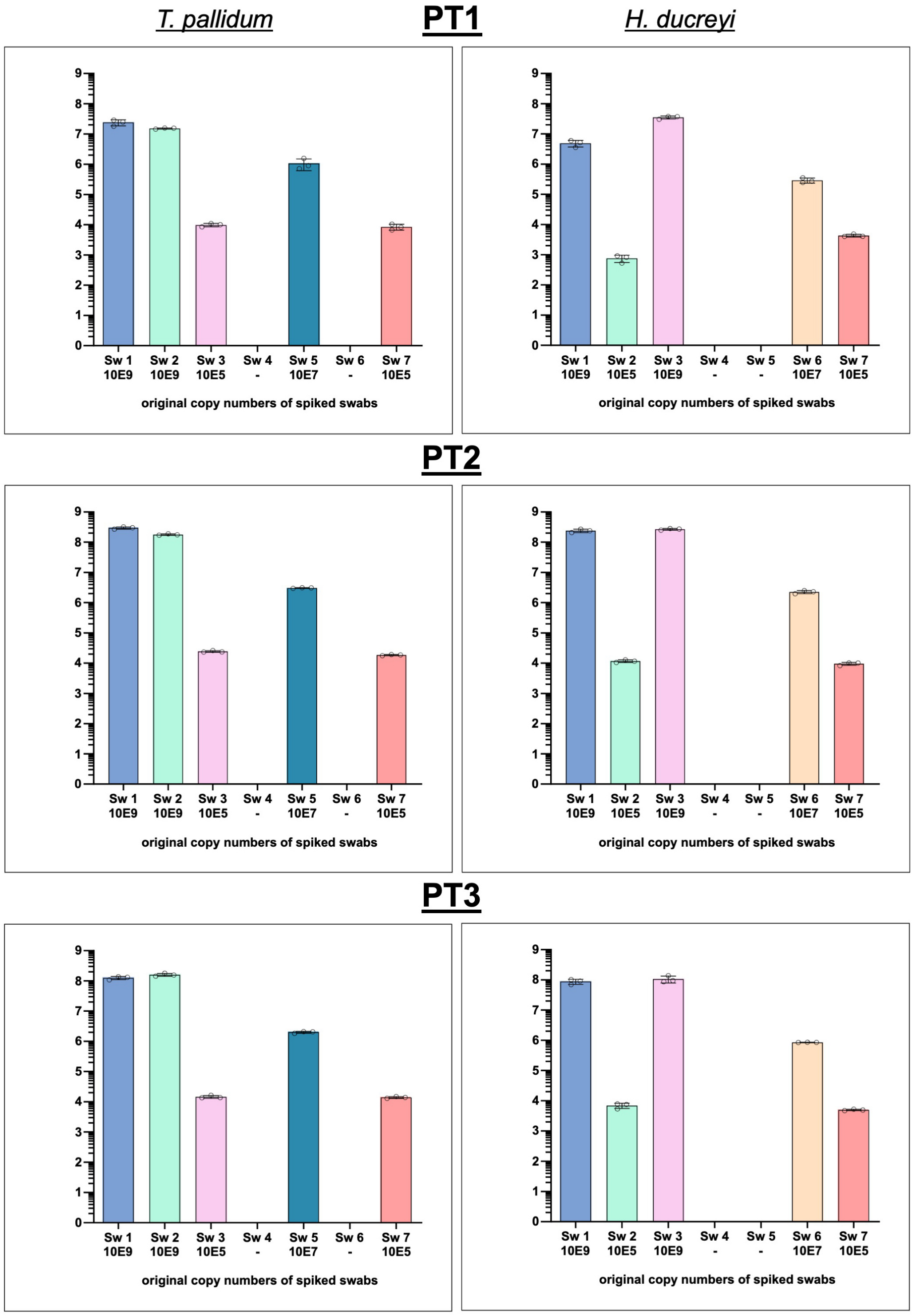

S2 Fig. Validation of the proficiency test items. The loss of copy numbers through proficiency test item extraction can be seen when the actual copy numbers for *TP polA* and *HD 16SrRNA* are compared to the original copy number shown below each bar. The PT numbers represent the round one to three. The bars represent the mean values, with the standard deviation values indicated. Samples were tested in triplicate (each measurement illustrated by a dot). The y-axis indicates the log-scale copy numbers, PT = proficiency test round, Sw = swab.
