# Supplementary material for: External quality assessment for yaws elimination in low- and middle-income countries using plasmid-based proficiency test items
Source: PLoS Negl Trop Dis. 2026 Mar 13;20(3):e0013772. doi: 10.1371/journal.pntd.0013772 (PMC13035232; doi:10.1371/journal.pntd.0013772)
Supplement: S1 Appendix — (PDF) [file pntd.0013772.s003.pdf]

Supporting Information

S1 Appendix

Determination of PT panel plasmid numbers

An overall two log-scale loss in copy numbers during DNA extraction was observed. To obtain post-extraction copy numbers that are comparable to field samples of skin lesions, swabs were spiked with 10^5 plasmids, followed by 10^7 (medium conc.) and 10^9 (high conc.) plasmids as two more PT differentiation steps. The test methods were run in single-plex (qPCR) or multi-plex (LAMP assay) format.

Cell cultivation

HEK293 (human embryonic kidney) cells were cultured in Dulbecco's Modified Eagle Medium (DMEM) + 10% FCS at 37°C. For harvesting, the cells were trypsinised, counted and resuspended in cell culture medium.

DNA extraction for qPCR

At FLI and the African RLs, DNA was extracted using the QIAamp Mini Kit (Qiagen). A 500 µl aliquot of lysis buffer was added to each tube containing the dry PTIs, and the contents of each tube were then mixed thoroughly. Next, 200 µl of the mixture were transferred to 1.5 ml-screw microtubes (Sarstedt Ref. No. 72.703.416) and subjected to DNA extraction in accordance with the manufacturer's protocol for "DNA purification from tissues." The DNA was eluted in two steps, with 50 µl of RNAse-free water in each step. Two 50 µl aliquots were obtained from each sample and stored at -20°C until further analysis.

DNA Extraction for LAMP assays

At the LSHTM and African DLs, DNA was extracted by using the Mast Isoplex DNA-RNA Extraction Kit (Mast Group GmbH, Germany). The tubes containing the dry PTIs were filled with 500 µl lysis buffer and mixed thoroughly. A total of 200 µl of sample lysis buffer was transferred to 200 µl of Lysis Solution V, along with 10 µl of carrier DNA, and placed into a 1.5 ml RNAse-free reaction tubes provided with the kit. The next step was to add 20 µl of Proteinase K and mix thoroughly. The samples were incubated at 56°C for one hour, with the samples being mixed several times during this period. After incubation, protocol was followed according to manufacturer's protocol but with incubation times of 30 sec instead of 20 sec for the single steps. Two 40 µl aliquots were obtained from each sample and stored at -20°C until further analysis.

Quantitative PCR

Three independent qPCR reactions (*RNAseP*, *TP*, *HD*) were used in this study. Sequences of primers and probes are shown in S3 Table. Reactions were generally run as triplicates except for the retesting of samples at the LSHTM. Briefly, the reaction mixes were prepared as follows: the *RNAseP* reaction consisted of 12.5 µl TaqMan FAST Advanced Master-Mix (Applied Biosystems), 1.25 µl of each primer (10 µM) and the probe (10 µM), 3.75 µl of RNAse free water and 5 µl template DNA.

The *TP polA* reaction consisted of 16.7 µl TaqMan FAST Advanced Master-Mix, 1.2 µl of each primer (25 µM) and 0.9 µl of the probe (5 µM) were prepared with 5 µl

template DNA. The *HD 16SrRNA* reaction consisted of 12.5 µl TaqMan FAST Advanced Master-Mix, 2.25 µl of each primer (10 µM) and the probe (10 µM), 3.75 µl of RNAse free water and 2 µl template DNA.

Cycling conditions are show in S4 Table. A no template control (NTC) containing nuclease-free water and a negative extraction control (extracted lysis buffer from QIAamp DNA Mini Kit, Qiagen) were included. Extracted DNA (in African RLs) for *RNaseP* and extracted DNA from *TP polA* or *HD 16SrRNA* gene target containing plasmids served as positive amplification controls. Plasmids served as a positive control. For quantification, a standard dilution series with plasmid DNA of *TP polA* or *HD 16SrRNA* in lambda DNA ranging from 10^6 to 10^1 copies was included. Due to the high copy numbers and purity of the plasmid DNA, the cut off for reporting positive PTI samples was set to Ct $\leq$35. qPCR was run on an Applied Biosystems 7500 real-time PCR system (ThermoFisher Scientific) in the African RLs or CFX96 real-time PCR system (BioRad) at FLI. Data were analyzed using QuantStudio software (ThermoFisher Scientific) or CFX Maestro software (BioRad), respectively.

Loop-Mediated Isothermal Amplification (LAMP)

LAMP assays were performed at the LSHTM and the participating African DLs. Primer sequences and the procedures of the assays are published elsewhere [1]. Samples were run using the MAST ISOPLEX Kit (MAST Diagnostica GmbH, Germany). Briefly, 18.5 µl reaction mix consisting of 5 µl reaction mix 5×, 5 µl primer mix, 0.5 µl dye mix and 8 µl nuclease free water was heated at 95°C for 10 min, then chilled on cool rack for 5 min. A 1.5 µl enzyme mix was added to the mixture. Subsequently, 5 µl template DNA was added. Samples were tested and analyzed with a MASTISOPLEX MD12 tube scanner (Mast Group) at 64°C for 60 min. All samples were measured as triplicates and categorised as positive or negative based on the manufacturer's algorithm. A no template control (NTC) containing nuclease free water and a negative extraction control (extracted lysis buffer from MAST ISOPLEX DNA/RNA Extraction Kit, Mast Group) were included as negative controls. Plasmids containing *TP polA* or *HD 16S* rRNA gene served as positive controls.

Validation of PTI robustness

In total, 22 FLOQSwabs (Copan) were spiked with 10^6 HEK293 cells and 10^7 *TP polA* or 10^7 *HD 16SrRNA* plasmid copies, respectively. Swabs were allowed to dry at environmental temperature in a laminar flow box and then inserted into 2 ml Eppendorf DNA LoBind reaction tubes (Eppendorf SE). The experiment was run in parallel for *TP* and *HD* plasmids. For baseline detection, DNA was extracted from a dry swab with a QIAamp Mini Kit (Qiagen). Next, three tubes were incubated at 56°C in a water bath at 80-100% humidity with DNA extracted after 3 h. The remaining tubes were incubated at 37°C with six swabs in a dry environment, six tubes in a moisture chamber with 80% humidity and six swabs in a wet environment with 50 µl RNAse free water added to each tube. Three tubes of DNA were extracted from each setting, one month and three months apart. The extracted DNA was stored at -20°C until it was measured by qPCR. All samples were tested in triplicate.

References

1. Becherer L, Knauf S, Marks M, Lueert S, Frischmann S, Borst N, von Stetten F, Bieb S, Adu-Sarkodie Y, Asiedu K, Mitjà O, Bakheit M. Multiplex Mediator Displacement Loop-Mediated Isothermal Amplification for Detection of

Treponema pallidum and *Haemophilus ducreyi*. Emerg Infect Dis. 2020;26(2):282–288.
