# Supplementary material for: External quality assessment for yaws elimination in low- and middle-income countries using plasmid-based proficiency test items
Source: PLoS Negl Trop Dis. 2026 Mar 13;20(3):e0013772. doi: 10.1371/journal.pntd.0013772 (PMC13035232; doi:10.1371/journal.pntd.0013772)
Supplement: S1 Table — (PDF) [file pntd.0013772.s004.pdf]

Supporting Information

S1 Table. Participating laboratories in the EQA programme of the LAMP4Yaws project.

Country	Health care	Institution	Test method performance
Ghana	National Reference Center	Noguchi Memorial Institute for Medical Research	qPCR
Ghana	District	Adeiso Health Center	LAMP
Ghana	District	Asuboi Health Centre	LAMP
Ghana	District	Tetteh Quarshie Memorial Hospital	LAMP
Côte d'Ivoire	National Reference Center	Institute of Pasteur Côte d'Ivoire	qPCR
Côte d'Ivoire	District	District Sanitaire de Yamoussoukro	LAMP
Côte d'Ivoire	District	Hôspital Général de Taabo	LAMP
Côte d'Ivoire	District	District Sanitaire de Divo	LAMP
Cameroon	National Reference Center	Centre Pasteur du Cameroun	qPCR
Cameroon	District	Centre médical d'arrondissement de Dimako	LAMP
Cameroon	District	Hôpital de District de Sangmelima	LAMP
Cameroon	District	Hôpital de District de Batouri	LAMP
Germany	Research Institution	Friedrich-Loeffler-Institut, Federal Research Institute for Animal Health	qPCR
UK	Research Institution	London School of Hygiene and Tropical Medicine	qPCR and LAMP
