# Supplementary material for: External quality assessment for yaws elimination in low- and middle-income countries using plasmid-based proficiency test items
Source: PLoS Negl Trop Dis. 2026 Mar 13;20(3):e0013772. doi: 10.1371/journal.pntd.0013772 (PMC13035232; doi:10.1371/journal.pntd.0013772)
Supplement: S2 Table — TP = Treponema pallidum; polA = polymerase A; HD = Haemophilus ducreyi. (PDF) [file pntd.0013772.s005.pdf]

Supporting Information

S2 Table. Gene target sequences inserted into the pMA vector. *TP* = *Treponema pallidum*; *polA* = polymerase A; *HD* = *Haemophilus ducreyi.*

Gene target	Insert sequence 5' – 3'
TP polA	ACGTACAGCAACGGTGGACGGTCATGCCAACACAATTACTTGATTTGTTCTCTCTCA<br>TGGGAGATTCCTCCGACAATGTGCCTGGTGTGAGAGGGATTGGTCCTAAGACGGCT<br>GCACATCTTCTCCACTGTTTTGGCACACTTGATGGTATTTATCGTCATACCTATTCCT<br>TAAAAGAAGCGCTGCGCACGAAGATAGTGTGTGGGAAGAAAGATGCATTTTTTTCTC<br>GTTCACTCATTGAGTTGCGTGACGATGTACCATGTGTTTTTTCGCTCGAAGATTCCT<br>GTTGTATTCCGCTCGATGTAACGTCTGCTGCACGTATTTTTGTGCGAGAAGGATTGC<br>ATGCGCTTGCACAACAATATCGTGCTTGTGTGCAAGAAATAGATACAGAAGCAACAA<br>ACGATACATTACAAATGACAGAGTCTTCTGTGCTCACGTCTGGTCGATGTGCAAATG<br>AGTGTTTCTTATCTCAGGTAGAAGGGAGGGCTAGTACACCGGAGGTGAACTCCGTAT<br>TGAAGTCGGAGTTGAAGACGAGTGCTGTGTCTGGCGCCATACCTATAGAAAATAGA<br>GATCTTAGGCAGGATGTTATGCTTGCACGCAGTGCAGGTCATTATCGTGGTGTTACT<br>GACCCTGTAGAACTTAAACGTATTATTGATTGCGCGTGTGCGAATGGTGTGGTCGCG<br>TTTGATTGTGAAACGGATGGATTGCATCCGCACGATACACGTCTGGTCGGATTTTCG<br>ATCTGCTTTCAGGAAGCAGAGGCTTTTTATGTTCCTCTTATTGTTCCGGACGTTTCT<br>CTTCATACCGAGTCAACTCAGTGTACATGTGCACGTAACACTAATGTCGAGACTGAA<br>AAGGAGTGCACAGAACAGCATGGGGTATCTGCATCTGCTGTGCAGGATCCGGCATAT<br>GTCCAAGCTGTCATGCACCAGCTTCGACGTCTTTGGAATGATGAGACGCTCACACTT<br>GTTATGCATAATGGAAAGTTTGATTATCACGTT
HD 16SrRNA	TGAGTTTGTGCCTTCGGGAACTATGTGACAGGTGCTGCATGGCTGTCGTCAGCTCGT<br>GTTGTGAAATGTTGGGTTAAGTCCCGCAACGAGCGCAACCCTTATCCTTTGTTGCCA<br>GCATGTAGTGATGGGAACTCAAAGGAGACTGCCAGTGATAAACTGGAGGAAGGTGG<br>GGATGACGTCAAGTCATCATGGCCCTTACGAGTAGGGCTACACACGTGCTACAATGG<br>CGTATACAGAGGGCGGCAAACCTGCAAAGGGGAGCGAATCTCACAAAGTACGTCTA<br>AGTCCGGATTGGAGTCT
