# Supplementary material for: External quality assessment for yaws elimination in low- and middle-income countries using plasmid-based proficiency test items
Source: PLoS Negl Trop Dis. 2026 Mar 13;20(3):e0013772. doi: 10.1371/journal.pntd.0013772 (PMC13035232; doi:10.1371/journal.pntd.0013772)
Supplement: S3 Table — TP = Treponema pallidum, HD = Haemophilus ducreyi, S = sense, AS = antisense, P = probe. (PDF) [file pntd.0013772.s006.pdf]

# Supporting Information

**S3 Table. Primer sequences used for qPCR and 3D digital PCR.** *TP* = *Treponema pallidum*, *HD* = *Haemophilus ducreyi*, S = sense, AS = antisense, P = probe.

| Target | Sequence 5' - 3' | Product size | Anneal. temp | Reference |
|---|---|---|---|---|
| *HD 16SrRNA* | **S:** TATACAGAGGGCGGCAAACC<br>**AS:** CCAATCCGGACTTAGACGTAC<br>**P:** 6-FAM-CAAAGGGGAGCGAATCTCAC-BHQ-1 | 65 bp | 60°C | [1] |
| *TP polA* | **S:** CAGGATCCGGCATATGTCC<br>**AS:** AAGTGTGAGCGTCTCATCATTCC<br>**P:** 6-HEX(FAM)-CTGTCATGC/BHQ1-dT/ACCAGCTTCG ACGTCTT | 71 bp | 60°C | [2] |
| *RNAse P* | **S:** CCAAGTGTGAGGGCTGAAAAG<br>**AS:** TGTTGTGGCTGATGAACTATAAAAGG<br>**P:** Cy5-CCCCAGTCTCTGTCAGCACTCCCTTC-BHQ-2(-3) | 80 bp | 60°C | [3] |
| M13 | **S:** GTAAAACGACGGCCAG<br>**AS:** CAGGAAACAGCTATGAC | variable | 55°C | [4] |

## References


1. Becherer L, Knauf S, Marks M, Lueert S, Frischmann S, Borst N, von Stetten F, Bieb S, Adu-Sarkodie Y, Asiedu K, Mitjà O, Bakheit M. Multiplex Mediator Displacement Loop-Mediated Isothermal Amplification for Detection of *Treponema pallidum* and *Haemophilus ducreyi*. Emerg Infect Dis. 2020;26(2):282–288.

2. Chen C-Y, Chi K-H, George RW, Cox DL, Srivastava A, Silva MR, Carneiro F, Lauwers GY, Ballard RC. Diagnosis of Gastric Syphilis by Direct Immunofluorescence Staining and Real-Time PCR Testing. J Clin Microbiol. 2006;44(9):3452–3456.

3. Tatti KM, Sparks KN, Boney KO, Tondella ML. Novel Multitarget Real-Time PCR Assay for Rapid Detection of *Bordetella* Species in Clinical Specimens. J Clin Microbiol. 2011;49(12):4059–4066.

4. Invitrogen. TOPO TA Cloning Kit Manual. Available from: `https://tools.thermofisher.com/content/sfs/manuals/topota_man.pdf` (last visited 02.04.2025).
