# Supplementary material for: External quality assessment for yaws elimination in low- and middle-income countries using plasmid-based proficiency test items
Source: PLoS Negl Trop Dis. 2026 Mar 13;20(3):e0013772. doi: 10.1371/journal.pntd.0013772 (PMC13035232; doi:10.1371/journal.pntd.0013772)
Supplement: S4 Table — FLI = Friedrich-Loeffler-Institut (provider), RL = Reference Laboratory. (PDF) [file pntd.0013772.s007.pdf]

Supporting Information

S4 Table. qPCR cycling conditions used by provider (FLI) and African Reference Laboratories for the detection of the (a) *RNAseP*, (b) *TP polA* and (c) *HD 16SrRNA* gene. FLI = Friedrich-Loeffler-Institut (provider), RL = Reference Laboratory.

(a)

RNAseP			**FLI**		**African RLs**	
**Step**	**Duration**	**Temp**	**Cycles**	**Cycler**	**Cycles**	**Cycler**
UNG incubation	2min	50°C	1	BioRad CFX96	1	ABI7500
DNA polymerase activation	10min	95°C	1		1	
Denaturation	15sec	95°C	40		45	
Elongation	30sec	60°C				

(b)

TP polA			**FLI**		**African RLs**	
**Step**	**Duration**	**Temp**	**Cycles**	**Cycler**	**Cycles**	**Cycler**
UNG incubation	2min	50°C	1	BioRad CFX96	1	ABI7500
DNA polymerase activation	30sec	95°C	1		1	
Denaturation	20sec	95°C	40		50	
Elongation	45sec	60°C				

(c)

HD 16SrRNA			**FLI**		**African RLs**	
**Step**	**Duration**	**Temp**	**Cycles**	**Cycler**	**Cycles**	**Cycler**
UNG incubation	2min	50°C	1	BioRad CFX96	1	ABI7500
DNA polymerase activation	10min	95°C	1		1	
Denaturation	15sec	95°C	40		40	
Elongation	1min	60°C				
