# Supplementary material for: External quality assessment for yaws elimination in low- and middle-income countries using plasmid-based proficiency test items
Source: PLoS Negl Trop Dis. 2026 Mar 13;20(3):e0013772. doi: 10.1371/journal.pntd.0013772 (PMC13035232; doi:10.1371/journal.pntd.0013772)
Supplement: S5 Table — PT = proficiency test, RL = reference laboratory. (PDF) [file pntd.0013772.s008.pdf]

Supporting Information

S5 Table. Summary of the qPCR test results from all three PT rounds (correctly identified/total). PT = proficiency test, RL = reference laboratory.

	RNAseP		***T. pallidum***		***H. ducreyi***	
	**RL Côte d'Ivoire**	**RL Cameroon**	**RL Côte d'Ivoire**	**RL Cameroon**	**RL Côte d'Ivoire**	**RL Cameroon**
PT1	7/7	7/7	7/7	7/7	7/7	7/7
PT2	7/7	7/7	7/7	7/7	7/7	7/7
PT3	7/7	7/7	6/7	7/7	7/7	6/7
**Total**	21/21	21/21	20/21	21/21	21/21	20/21
**Performance %**	100%	100%	95%	100%	100%	95%
