# Supplementary material for: External quality assessment for yaws elimination in low- and middle-income countries using plasmid-based proficiency test items
Source: PLoS Negl Trop Dis. 2026 Mar 13;20(3):e0013772. doi: 10.1371/journal.pntd.0013772 (PMC13035232; doi:10.1371/journal.pntd.0013772)
Supplement: S6 Table — TP = Treponema pallidum; HD = Haemophilus ducreyi; pos = positive; neg = negative. (PDF) [file pntd.0013772.s009.pdf]

Supporting Information

S6 Table. Overview of the DNA extracts from field samples that were retested at the London School of Hygiene & Tropical Medicine. *TP* = *Treponema pallidum*; *HD* = *Haemophilus ducreyi*; pos = positive, neg = negative.

**Participants**	**Total**	***TP* pos**	***TP* neg**	***HD* pos**	***HD* neg**	**Samples retested (20% *TP* pos)**	**Samples retested (20% *TP* neg)**	**Correct ident. (%)**
Côte d'Ivoire	98	25	73	14	84	5	15	20/20 (100%)
Cameroon	24	18	6	0	24	4	1	5/5 (100%)
